# Head motion synchrony in unidirectional and bidirectional verbal communication

**DOI:** 10.1371/journal.pone.0286098

**Published:** 2023-05-24

**Authors:** Jinhwan Kwon, Hiromi Kotani

**Affiliations:** Department of Education, Kyoto University of Education, Kyoto, Japan; New York University Abu Dhabi, UNITED ARAB EMIRATES

## Abstract

Interpersonal communication includes verbal and nonverbal communication. Verbal communication comprises one-way (e.g., a speech or lecture) and interactive verbal communication (e.g., daily conversations or meetings), which we frequently encounter. Nonverbal communication has considerable influence on interpersonal communication, and body motion synchrony is known to be an important factor for successful communication and social interaction. However, most research on body motion synchrony has been elucidated by either the setting of one-way verbal transmission or the verbal interaction setting, and it remains unclear whether verbal directionality and interactivity affect body motion synchrony. One-way and two-way (interactive) verbal communication is implicated in designed or undesigned leader–follower relationships, and also in the complexity and diversity of interpersonal interactions, where two-way verbal communication is more complex and diverse than in the one-way condition. In this study, we tested head motion synchrony between the one-way verbal communication condition (in which the roles of the speaker and listener are fixed) and the two-way verbal communication condition (where the speaker and listener can freely engage in a conversation). Therefore, although no statistically significant difference in synchrony activity (relative frequency) was found, a statistically significant difference was observed in synchrony direction (temporal lead-lag structure as mimicry) and intensity. Specifically, the synchrony direction in two-way verbal communication was close to zero, but this in one-way verbal communication was synchronized with the listener’s movement predominantly delayed. Furthermore, synchrony intensity, in terms of the degree of variation in the phase difference distribution, was significantly higher in the one-way verbal communication than in the two-way condition, with bigger time-shifts being observed in the latter. This result suggests that verbal interaction does not affect the overall frequency of head motion synchrony but does affect the temporal lead-lag structure and coherence.

## Introduction

Interpersonal communication is indispensable for daily living and social life and has various roles such as the function of transmitting information, building human relationships, and sharing emotions in society [1, 2]. Interpersonal communication includes verbal and nonverbal communication. Verbal communication conveys information such as thoughts, feelings, and intentions through spoken and written words [2, 3], and includes one-way (such as a speech or lecture) and interactive verbal communication (such as daily conversations or meetings) that we frequently encounter [2, 3]. Meanwhile, nonverbal communication is characterized by non-linguistic forms inferred from the aspects of informative behavior (facial expressions, gaze, and gestures) and paralanguage (prosody, pitch, volume, and intonation) [4, 5]. Nonverbal communication has a substantial influence on interpersonal communication and is recognized as an important factor in successful communication and social interaction [6–8]. Accordingly, nonverbal behavior is attracting attention as a tool that can be used to objectively evaluate the state of communication—such as methods for estimating emotions through facial expressions and measuring attention and feedback through eye contact and gaze [9–13]. In particular, head movement (movement accompanying utterances and backchannel communication) is regarded as a form of universal nonverbal behavior that occurs frequently during the communication process and a channel through which the speaker and the listener interact in real time [14–18]. This study focuses on the relationship between verbal and nonverbal communication, with particular focus on head movement.

In the field of communication science, many researchers have verified that the characteristics of interaction in human communication are manifested by the synchrony phenomenon of body movements [19–35]. Body motion synchrony represents the temporal coordination of body movements during the communication process, and many researchers have reported that bodily synchrony in interpersonal communication is exhibited in diverse relationships and contexts, such as between a mother and infant [19–22], musicians in music ensembles [23], physician and patient [24], and psychological counsellor and client [25]. Body motion synchrony is also correlated with positive effects on interpersonal communication, such as enhancing affiliation [26], rapport [27], bonding [28], and empathy [29, 30], as well as fostering cooperation [31] and good impression [32]. Specifically, Fujiwara et al. reported that rhythmic features of body motion synchrony contribute to bonding between individuals [28], while Hove and Risen concluded that interpersonal synchrony leads to affiliation, and the degree of synchrony predicts subsequent affiliation ratings [26]. In addition, synchrony entailed affect wherein positive affect was found to be associated positively with synchrony [33], and higher nonverbal synchrony (characterized as psychotherapies) was linked to higher symptom reduction [34]. However, most research on body motion synchrony has been elucidated by either the setting of one-way verbal transmission (as in speech) or the verbal interaction setting (as in free conversation) [24–37]. The current study focused on the effects of one-way and two-way (interactive) verbal communication on body motion synchrony in interpersonal communication.

Traditionally, the roles in movement synchrony have been verified as the forms of generator and receiver on the basis of entrainment [38–43]. This concept has been extended to the roles of leaders and followers in interpersonal synchronization [44–46]. In simple experimental environments such as finger tapping, the mechanisms of the leader–follower relationship have been clearly shown by a lagged cross-correlation analysis, even when the roles were not predetermined [44, 45]. According to a recent study, when a leader role (which involves providing a tapping sound) is predetermined in joint tapping, the other party naturally becomes a follower [46]. The abovementioned studies show that the roles of leader and follower are essential for body motion synchrony, and that simple, repetitive and predictable movements facilitate human synchronization during nonverbal interaction [44–47]. Another recent approach is the mirror game, which characterizes nonverbal interaction while mirroring the other’s movements [48]. In particular, Ravreby et al. reported that, when playing the mirror game, the vast majority of the dyads did not consistently play the role of leader or follower, but rather changed roles during the game [35]. Furthermore, although simple, repetitive, and predictable movements benefit from greater synchronization, more complex and novel movements are preferred to increase mutual interest and experience better interaction [35]. One-way verbal communication will align in a situation where a leader–follower relationship is set, and two-way (interactive) verbal communication will be associated with a situation where there is no leader–follower design [2, 3, 49]. In addition, two-way verbal (interactive) communication is more complex and diverse than one-way verbal communication, because, in the former, the communicators need to predict speech timing, request and perform turn-taking, and encode messages as linguistic or nonverbal information simultaneously during interactive verbal communication [2, 3, 50, 51].

To reiterate the above, body motion synchrony has been elucidated by either the setting of one-way verbal transmission or the two-way (interactive) conversation setting [24–37], and it remains unclear whether verbal directionality and interactivity affect body motion synchrony. Regarding verbal communication, one-way and two-way verbal communication will be implicated in designed or undesigned leader–follower relationships, and in the complexity and diversity of interpersonal interaction [2, 3, 35, 49–51]. In this study, we hypothesized that the characteristics of head motion synchrony would vary depending on one-way or two-way verbal directions in interpersonal communication. We set the following two types of communication conditions based on the face-to-face communication format: (1) a one-way verbal communication condition in which the roles of the speaker and listener are fixed, and (2) a two-way (interactive) verbal communication condition in which the roles of the speaker and listener are not fixed and they freely change the roles through free conversation. We focused on head movements taking place in the two communication patterns, which constantly occur in the process of interpersonal communication in the forms of the speaker’s utterance and the listener’s backchanneling [14–18]. In addition, we detected the phase difference distribution to uncover differences between the two types of verbal communication patterns.

## Materials and methods

### Experimental designs

In the one-way verbal communication condition, the speaker conveys certain content to the listener in a face-to-face state. The listener cannot ask questions or speak but can freely interact through backchannel signals, including brief words or through nonverbal cues such as facial expressions and head nods. In the two-way (interactive) verbal communication condition, the roles of the speaker and listener are not fixed during the face-to-face communication process and they can freely engage in a conversation, exchange turns while speaking, and interact using nonverbal signals. As degrees of interpersonal relationships affect body motion synchrony [26–28], in both conditions, the pairs of participants were defined in the first encounter without any interpersonal relationship. A small accelerometer was directly attached to the participant’s forehead; a set of phase differences were extracted from the time-series data on the acceleration, and head motion synchrony was analyzed through the phase difference distribution.

### Participants

In total, 48 participants (24 males, 24 females, mean age 21 years) were divided into two groups; 24 respondents were assigned to the one-way verbal communication condition, and 24 were assigned to the two-way condition. In the experiment, we imposed restrictions for participation, and so the respondents had to meet the following requirements: a pair of individuals of the same sex who met for the first time, having an age difference of less than three years, and having Japanese as their native language. All participants had normal or corrected-to-normal vision and normal auditory perception. No participant had any prior knowledge of, or experience with the experiment. Experimental procedures were approved by the local Ethical Committee of the Kyoto University of Education in accordance with the guidelines of the Declaration of Helsinki for research involving human participants. All respondents provided their written informed consent prior to engaging in the experiment.

### Apparatuses

**Fig 1:**
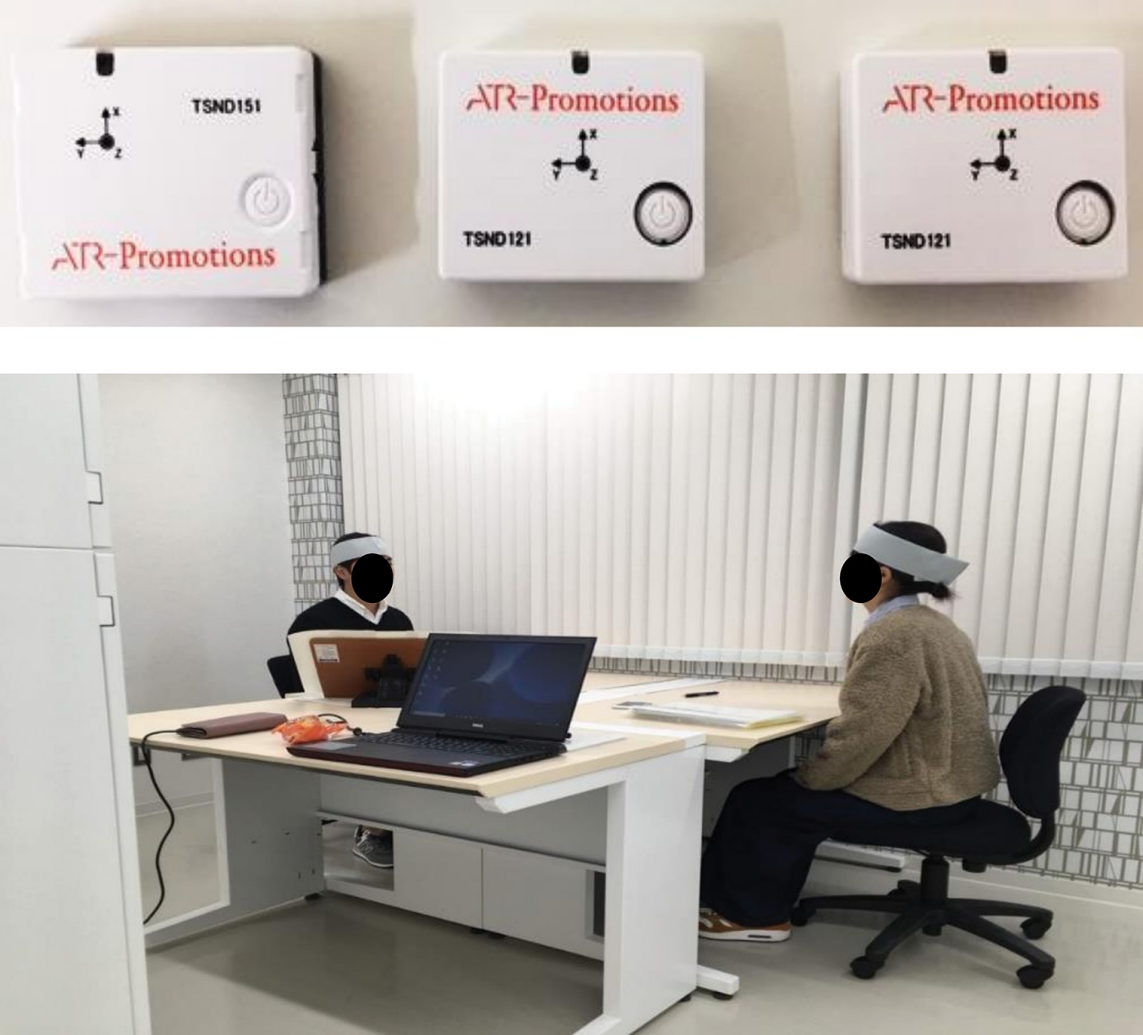
Schematic illustration of the experiment. Participants sat at a table and conducted the face-to-face communication experiment. Three axis acceleration sensors were used in our experiment.

Fig 1 provides an illustration of the experiment. Participants sat at a table, 1.8 m apart from each other, and conducted the face-to-face communication experiment without face masks. The temperature of the room was 20.2°C, while the illuminance was 1,432 lx (CANA-0010, Tokyo Photoelectric, Japan), and the environmental noise was 31.4 dB (CHE-SD1, Sanwa Supply, China). Three axis acceleration sensors were used in our experiment. Moreover, two accelerometers (TSND121, ATR-Promotions, Japan) were employed to record the head movements of each participant, and another accelerometer (TSND151, ATR-Promotions, Japan) was used to calculate the start and end of the experiment to synchronize the data of the two participants in time. We acquired time-series data via Bluetooth on a PC (Inspiron 15 7000, Dell, TX, USA) by attaching a three-axis acceleration sensor to the participant’s forehead. The acceleration sensor was small enough to attach to the forehead (37 mm(W) × 46 mm(H) × 12 mm(D)), weight 22g). The sampling frequency for acquiring time-series data was set to 100 Hz. In addition, a video camera (HDC-TM45, Panasonic, Japan) was used to record the participant’s movements and conversational voice throughout the experiment.

### Experimental procedures

In the one-way verbal communication condition, the roles of the speaker and the listener were predetermined before the experiment commenced, and the participants sat facing each other across the table. A Wikipedia article entitled “cashless society” was shared with the speaker in advance, and the speaker practiced describing the article to the experimenter in their own words. Those who were familiar with this content were excluded from the experiment. The article comprised a total of 2,599 characters, which was sufficient to provoke discussion for approximately 10 min. The speaker was instructed to speak in a clear audible voice, and the speaker and listener were instructed to look at each other. The listener was instructed to listen carefully to what the speaker was saying and was unable to ask questions, but was allowed to use backchannel behaviors such as facial expression, nodding, and brief words—including "un", "hai", and "ee,” which are equal to “mmhm,” “uh huh”, and “yeah” in English [15–18]. The experimenter presented the cues of the start and end of the experiment to the participants. Subsequently, the temporal information of the cues was recorded on the PC through the acceleration sensor.

In the two-way verbal communication condition, a free conversation was conducted in which both participants could freely talk, exchange turns while speaking, and interact using nonverbal signals. As examples of conversation content, we provided some direction along the lines of self-introduction, hobbies, and how to spend the weekend, which are typical topics spoken at a first meeting quite naturally without any trouble. The conversation lasted approximately 10 min, and the experimenter intervened at an appropriate point to end the experiment. Just as was the case in the one-way verbal communication condition, the experimenter presented the cues of the start and end of the experiment to the participants. Following this, the temporal information of the cues was recorded on the PC through the acceleration sensor.

### Data analysis

#### Detection of phase difference

The phase difference based on the acceleration data is used in the synchrony detection method and the algorithm of Kwon was adopted for the phase difference detection [49]. The three -axis (XYZ) time-series data correspond to frontal, horizontal, and sagittal plane motions. Here, the norm data *a*(*t*_*i*_) of the obtained accelerations were calculated as

$$a(t_{i})=\sqrt{a_{x}{}^{2}(t_{i})+a_{y}{}^{2}(t_{i})+a_{z}{}^{2}(t_{i})}\;\mathrm{for}\;i=0,1,2,fori=0,1,2,\ldots .$$ (1)

Since the sampling frequency is 100 Hz, *t*_*i*_ and *t*_*i*+1_ are obtained with an interval of 10 ms. The amplitudes of the head movement caused by speech rhythm, utterance, or nodding are different between the individuals. Therefore, the norm data *a*(*t*_*i*_) was normalized so as to avoid it being affected by the absolute amplitudes of the movement.


$$a^{\prime }(t_{i})=\frac{a(t_{i})-\bar{a}}{\sigma_{a}}.$$ (2)

Here, $\bar{a}$ and *σ*_*a*_ are calculated as

$$\bar{a}=\sum_{t_{i}\in T}\frac{a(t_{i})}{\left|T\right|},$$ (3)

$$\sigma_{a}=\sqrt{\frac{\sum_{t_{i}\in T}{\left(\bar{a}-a(t_{i})\right)}^{2}}{|T|-1}},$$ (4)

where *T* represents the total measurement time in each condition. Subsequently, to reduce the fluctuation due to the noise of the signal, smoothing was performed with a moving average of 100 ms. We calculated the time-series data *a*′(*t*_*i*_) as follows:

$$\bar{a^{\prime }}(t_{i})=\frac{1}{11}\sum_{l=i}^{i+10}a^{\prime }(t_{l})\;\mathrm{for}\;i=0,1,2,\ldots .$$ (5)

Head movements associated with speech rhythm, utterance, or nodding have the characteristic of periodic motion. Therefore, when head movement occurs, there is a maximum value, termed a peak, in the time series data. In this study, we focused on the value of this peak and used it for phase difference calculation. The peak of acceleration $\bar{a^{\prime }}(t_{i})$ is defined as

$$\bar{a^{\prime }}(t_{i})-\bar{a^{\prime }}(t_{i\pm 1})>0.$$ (6)

To extract head movements that are reliable for synchronization phenomena, the amplitude threshold was set to 2.0 [49], and peaks of 2.0 or more were extracted. Thus, we imposed the following conditions on $\bar{a^{\prime }}(t_{i})$:

$$\bar{a^{\prime }}(t_{i})-2.0\geq 0.$$ (7)

The phase difference is defined as the minimum temporal difference between the peaks of the participants, while the maximum delay time of synchrony is defined as 1.0 s [49]. Therefore, the phase difference in this study was calculated as the minimum temporal difference (*t*_*j*_ − *t*_*i*_) from the peak time (*t*_*j*_) of participant A to that (*t*_*i*_) of participant B after detecting the peak of the acceleration data of each participant.


$$-1.0s\leq t_{j}-t_{i}\leq 1.0s.$$ (8)

#### Synchrony analysis

We used the synchrony analysis method of Kwon for characterizing body motion synchrony [49], using four statistical measurements based on the distribution of phase differences obtained from the detection algorithm. The four statistical measurements included density, mean phase difference, standard deviation (SD), and kurtosis. Density refers to the relative frequency that is calculated as the frequency of phase difference per minute within each pair, which represents the *synchrony activity*. Mean phase difference refers to the mean value of the phase difference distribution and indicates whose body movement led or delayed the synchrony during communication. Therefore, mean phase difference represents the *synchrony direction* as the temporal lead-lag structure in body motion synchrony. Furthermore, SD and kurtosis represent the *synchrony intensity* in body motion synchrony; SD represents the degree of variation in the phase difference distribution, and kurtosis represents the degree of convergence to the mean in the phase difference distribution. Synchrony intensity in theoretical research is based on a phase difference of 0 ms, termed as the perfect synchronization, and the closer the phase difference is to 0, the higher the intensity [39–42]. Even in face-to-face communication, the stronger the motion synchrony, the smaller the spread width (SD) of the phase difference distribution, and the higher the degree of convergence to the mean.

## Results

**Fig 2:**
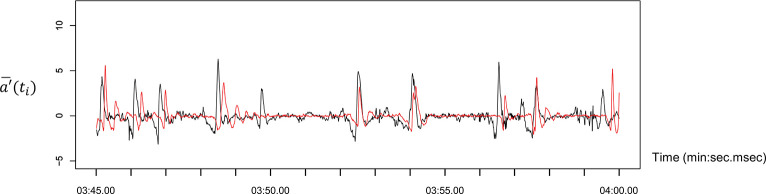
Typical time-series data for head movement in the one-way verbal communication condition. The black line shows the Speaker’s acceleration data, and the red line indicates the listener’s acceleration data.

**Fig 3:**
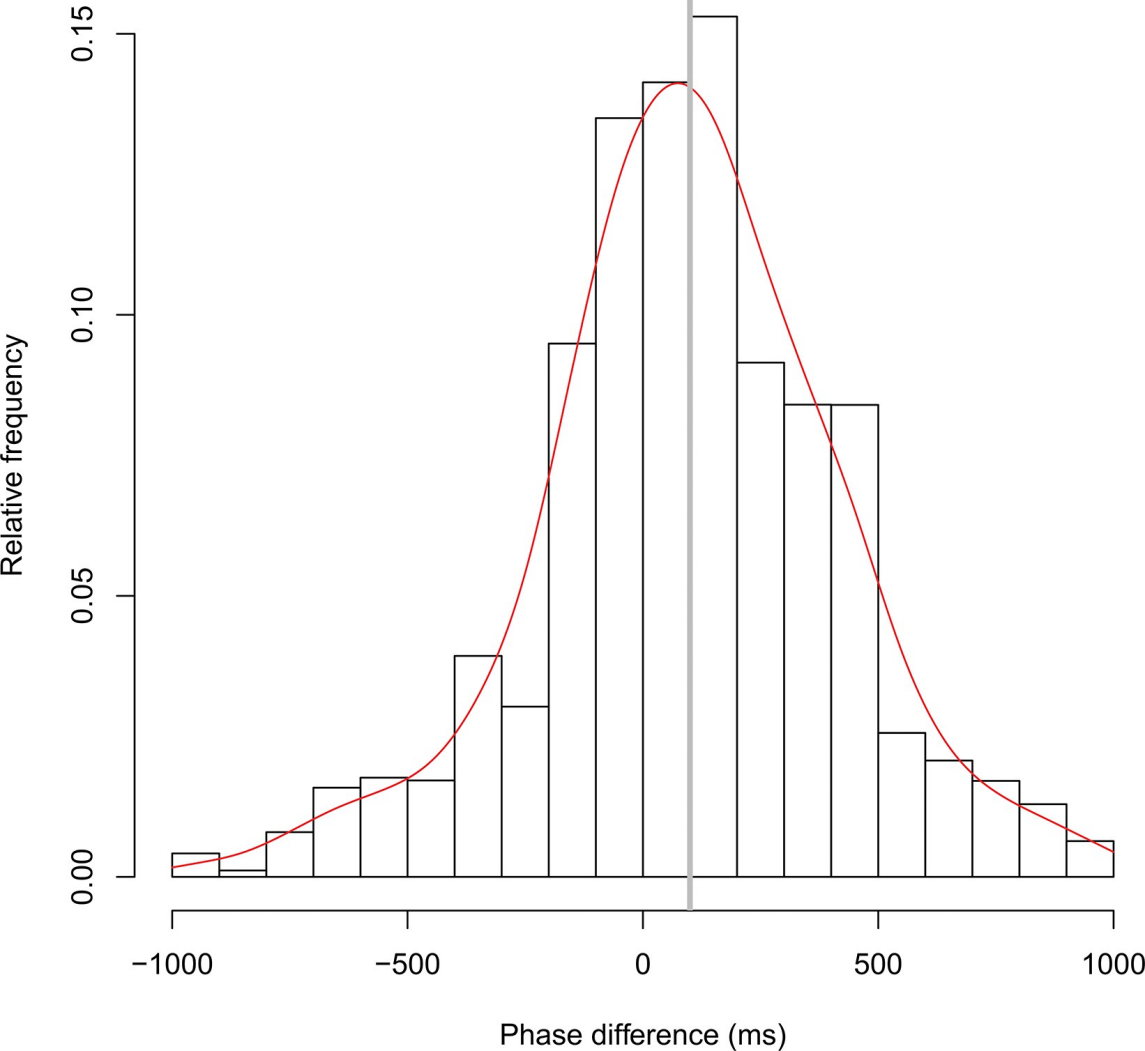
Total results from the one-way verbal communication condition. Distribution of the phase difference in all the pairs in the one-way verbal communication condition. The horizontal axis represents the phase difference between participants; a positive value indicates that the listener’s head movement occurs later than that of the speaker, and a negative value indicates that the speaker’s head movement occurs later than that of the listener. The vertical axis represents the relative frequency in the 100 ms interval class of the phase difference distribution. The gray vertical bar represents the mean of the overall phase difference distribution, and the red line is fitted by the smoothing spline curve in each class of the distribution.

**Fig 4:**
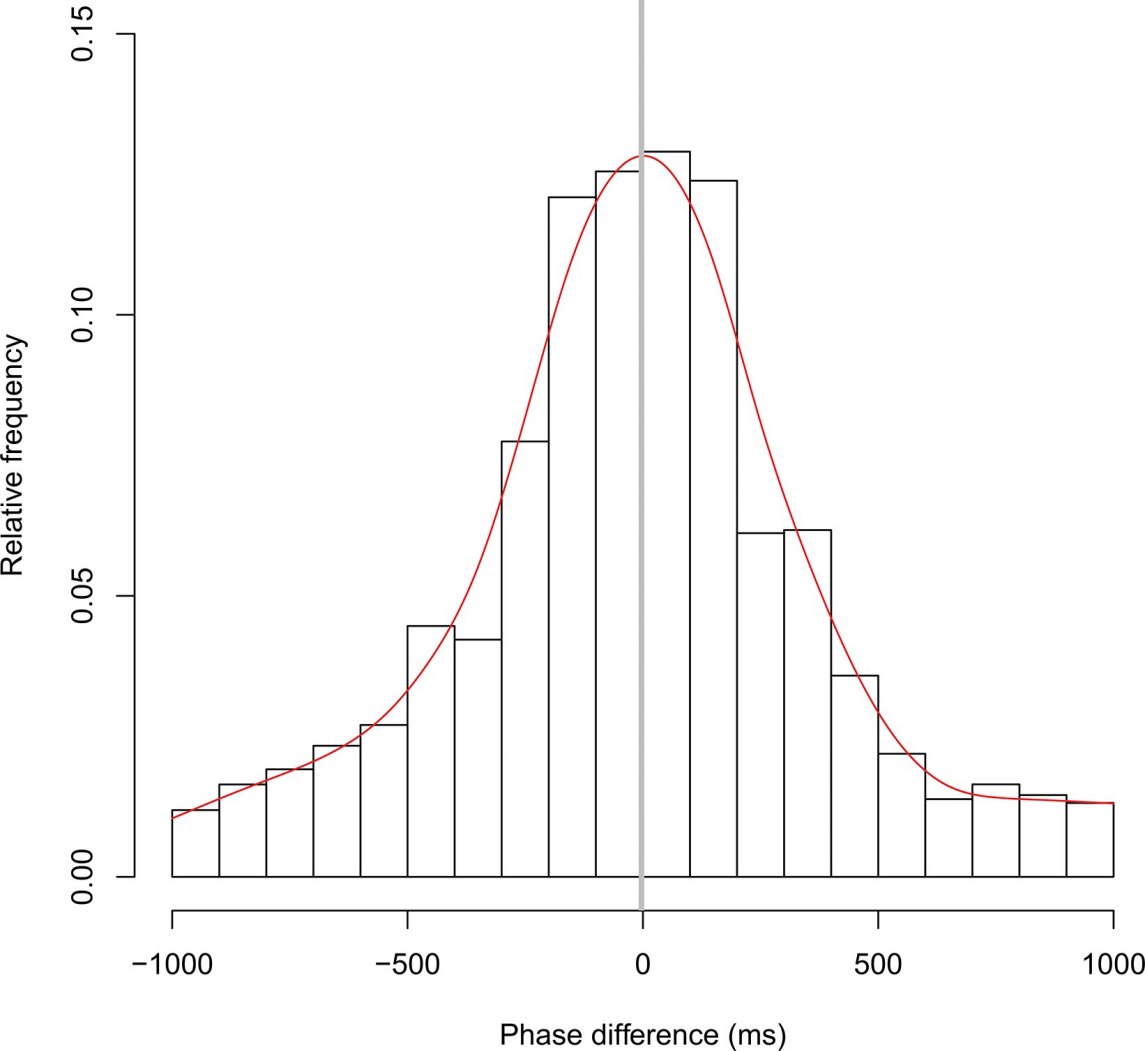
Total results from the two-way verbal communication condition. Distribution of the phase difference in all the pairs in the two-way verbal communication condition. The description of Fig 4 was the same as in Fig 3, but a positive value indicates that either of the participant’s movements tends to delay the other participant’s movements and vice versa.

Fig 2 shows typical time series data for head movements observed during the experiment. Fig 3 illustrates the distribution of the phase difference in all the pairs in the one-way verbal communication condition (see also S1 Fig). The horizontal axis represents the phase difference between participants; a positive value indicates that the listener’s head movement occurs later than that of the speaker, and a negative value indicates that the speaker’s head movement occurs later than that of the listener. The vertical axis represents the relative frequency in the 100 ms interval class of the phase difference distribution. The gray vertical bar represents the mean of the overall phase difference distribution, and the red line is fitted by the smoothing spline curve in each class of the distribution. Fig 4 demonstrates the distribution of the phase difference in all the pairs in the two-way verbal communication condition (see also S2 Fig). The description of Fig 4 is the same as that of Fig 3, but a positive value indicates that either of the participant’s movements tends to delay the other participant’s movements and vice versa.

In the one-way verbal communication condition, the mean density was 8.8 times/min (SD = 2.4), and the overall mean of the mean phase differences was 100 ms (SD = 66 ms, see also S1 Table). The mean of the SDs was 310 ms (SD = 50 ms) and the mean of the kurtosis was 0.9 (SD = 0.9). In the two-way verbal communication condition, the mean density was 8.6 times/min (SD = 2.5), and the overall mean of the mean phase differences was −3 ms (SD = 43 ms). The mean of the SDs was 370 ms (SD = 69 ms) and the mean of the kurtosis was 0.3 (SD = 0.6).

**Fig 5:**
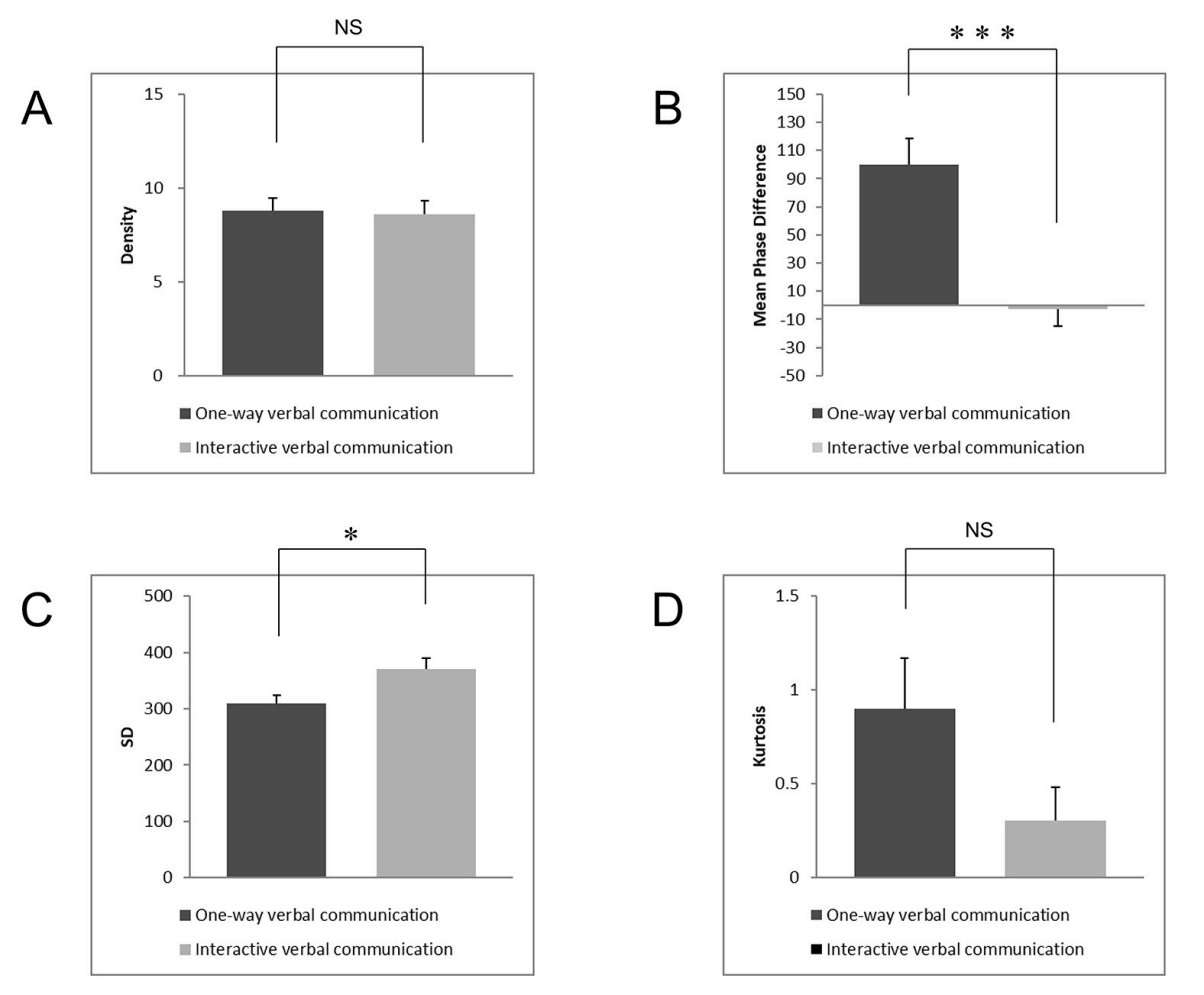
Comparison of results between both conditions. (A) indicates the mean density, (B) indicates the mean phase difference, (C) indicates the mean SDs and (D) indicates the mean kurtosis in the phase difference distribution in each condition, respectively. The error bars show the standard errors in each condition and indicator, *: *p* < .05, **: *p* < .01, ***: *p* < .001 (two-tailed, independent t-test).

The two-tailed independent t-test, Cohen’s d, and post hoc power analyses were used to analyze the data. There was no significant difference in density between both conditions (t (22) = 0.135, p = 0.894, d = 0.055, post hoc power = .052; see Fig 5A). However, the mean phase difference was significantly delayed in the one-way verbal communication group (mean = 100, SD = 66) compared to the two-way verbal communication group (mean = −3, SD = 43), (t (22) = 4.479, p = 0.0002, d = 1.828, post hoc power = .989; see Fig 5B). Moreover, SDs were significantly higher in the two-way verbal communication group (mean = 370, SD = 69) compared to the one-way verbal communication group (mean = 310, SD = 50), (t (22) = −2.234, p = 0.036, d = 0.912, post hoc power = .569; see Fig 5C). However, there was no significant difference in the kurtosis (t (22) = 1.848, p = 0.078, d = 0.754, post hoc power = .424; see Fig 5D). This result means that, regarding the two intensity measures, the two-way verbal communication group showed greater variation in the phase difference distribution than did the one-way verbal condition, but there was no kurtosis difference, that is, no difference in the degree of convergence to the mean in the phase difference distribution.

## Discussion

In this study, we investigated the characteristics of head motion synchrony in the contexts of one-way and two-way (interactive) verbal communication through phase differences in interpersonal communication. In previous studies, body motion synchrony has been elucidated by either the setting of one-way verbal transmission (as in speech) or the verbal interaction setting (as in free conversation). However, it was not clear whether verbal directionality and interactivity affect body motion synchrony. Throughout the two types of conditions assessed in the present study, head motion synchrony was characterized as activity, direction, and intensity using four statistical indicators (density, mean phase difference, SD, and kurtosis) based on the phase difference distribution [49]. As a result, although no statistically significant difference was observed in synchrony activity, a statistically significant difference was found in synchrony direction and synchrony intensity. In particular, the synchrony direction in two-way verbal communication was close to zero, but the synchrony direction in one-way verbal communication tended to be synchronized with the listener’s movement predominantly delayed. Additionally, in terms of the degree of variation in the phase difference distribution, the synchrony intensity was significantly higher in the one-way verbal communication than that in the two-way condition, with bigger time-shifts being observed in the latter. This suggests that while verbal interaction does not affect the overall frequency of head motion synchrony, it does affect temporal lead-lag structure and coherence. Referring to the above result, we now discuss the characteristics of head motion synchrony between one-way and two-way (interactive) verbal communication.

No significant difference was found in synchrony activity between one-way verbal communication (8.8 nods/min) and interactive verbal communication (8.6 nods/min). This suggests that verbal directionality and interactivity do not affect the overall frequency of head motion synchrony; the aforementioned finding could be attributed to the mechanism of consistent and continuous feedback as a function of nonverbal behavior, which has been regarded as an important factor in interpersonal communication [4, 5, 14–17]. Interpersonal feedback encompasses all forms of human communication: verbal and nonverbal (e.g., behavior, movement, and paralanguage as prosody, pitch, volume, and intonation) [2–5]. In particular, the frequency of the listener’s response is well defined in terms of the backchannel, and the backchannels, including nonverbal gestures (nods and smiles), nonverbal vocalizations (mm, uh-huh, laughs), and verbal expressions (yes, right), constitute an important aspect of performance enhancement and promotion of interest levels [15–18]. Moreover, backchannel cues involve responsive feedback to the speaker to provide information about the addressee’s ongoing engagement in the dialogue [52]. Despite some cultural differences, vocal responses as a form of backchanneling were found to occur at the rate of 6.1 times/min (calculated from the results of White [17]), and head nods co-occurred with vocalized responses approximately 85% of the time [17]. This result is consistent with Dittmann and Llewellyn’s finding, which also demonstrated that visual and auditory backchannels tend to occur at roughly the same points [53]. However, several other non-verbal behaviors including non-vocalized head nods, shakes, and gestures also occur and interact through the feedback channel [4, 5, 14–17]. This consistent and continuous feedback and interaction through various channels is an essential factor for continuous and successful communication [54–56]. Therefore, perhaps the total amount of head motion synchrony did not change due to this consistent and continuous feedback property, regardless of verbal directionality and interactivity.

In this study, the synchrony direction was 100 ms for one-way verbal communication and −3 ms for interactive verbal communication, showing a statistically significant difference. From the synchrony direction, we could confirm the specific participants who led the movement synchrony. In one-way verbal communication, the listener tended to synchronize with a time lag, but in interactive verbal communication, the time lag tended to be close to zero. This result expresses, fairly well, the characteristics of verbal interactivity and directionality. In one-way verbal communication, because the roles of the speaker and the listener are fixed and verbal information is always transmitted from the speaker to the listener, it is highly likely that the speaker played the role of the leader providing information. However, the listener might play the role of the follower giving passive feedback on the speaker’s information delivery. According to previous studies, it is known that the listener’s nods occur in accordance with the speaker’s utterance rhythm [57]. In addition, according to Stivers, nods by a listener act as a sign of alignment with the activity of speaking and affiliation through a claim of access to the speaker’s stance, either indirectly or directly [58]. Consequently, the synchrony direction in one-way verbal communication may be delayed for the listener who receives the verbal information. However, regarding this study, in the interactive verbal communication condition, the synchrony direction was close to zero. In interactive verbal communication, the speaker and the listener were not fixed, and the roles were alternated at any time. This correlated with the fact that the roles of both leaders and followers were swapped in communication. Such exchanges in speaking turns may have particular outcomes: the mean phase difference becomes close to zero in interactive verbal communication, unlike that in one-way communication.

A statistically significant difference in synchrony intensity, in terms of the degree of variation, was noted between the two kinds of communication, with the time-shifts being bigger in interactive verbal communication than in the one-way condition. The smaller the time shift, the more the phase difference tends not to become dispersed, and the higher the temporal coherence. Because the roles of the speaker and listener are fixed in one-way verbal communication, they can concentrate on each role, and stable physical interaction occurs during the communication process. That is, the speaker continues the head movement associated with the utterance to convey the message, and the listener continues the head movement associated with the backchannel. Therefore, this stable performance and interaction in communication processing may be attributed to contexts where the temporal coherence becomes high.

Regarding interactive verbal communication, the bigger time shift seems to be caused by more complex and integrated processing in communication. Contrary to one-way verbal communication, the communicator needs to predict speech timing, request and perform turn-taking, and encode messages as linguistic or nonverbal information in interactive verbal communication [50, 51, 59–62]. In other words, there are more types of information and behaviors to be accompanied and processed simultaneously in interactive verbal communication than there are in one-way verbal communication. Additionally, one who becomes a listener provides feedback to the speaker while decoding and interpreting the received messages, predicting the timing of the turn-taking process, and simultaneously composing and transmitting the messages [50, 51, 61, 62]. Moreover, they should change topics, convey and accept new conversational content, and provide appropriate nonverbal feedback for continuous and successful communication [2–8, 14–18, 52, 53]. Ravreby et al. reported that higher levels of complexity and novelty in nonverbal interaction are introduced to keep each participant interested and engaged [35]. Therefore, such factors, which are more complex and diverse in interactive verbal communication, lead to lower temporal coherence.

To summarize, we tested head movement synchronization during one-way verbal communication versus mutual verbal communication. There was almost no time delay in the mutual verbal communication, whereas there was a remarkable delay in the listeners’ movements in the one-way verbal communication. However, with a larger delay, the synchronization intensity was higher in one-way verbal communication compared to mutual verbal communication. This finding shows that verbal interaction does not affect the overall frequency of head motion synchrony but does affect temporal lead/delay and coherence. Because we used different topics in both conditions, in future, we need to investigate whether the different topics affect body motion synchrony. At the same time, it is also necessary to increase the sample size.

## Supporting information

S1 Fig Distribution of the relative frequency of synchronized head nods for each pair in the one-way verbal communication condition. (TIF)

S2 Fig Distribution of the relative frequency of synchronized head nods for each pair in the interactive verbal communication condition. (TIF)

S1 Table Results of the one-way and two-way verbal communication conditions. (PDF)
