# Supplementary material for: Head motion synchrony in unidirectional and bidirectional verbal communication
Source: PLoS One. 2023 May 24;18(5):e0286098. doi: 10.1371/journal.pone.0286098 (PMC10208465; doi:10.1371/journal.pone.0286098)
Supplement: S1 Table — (PDF) [file pone.0286098.s003.pdf]

S3 Table

Table 1. Result of the one-way verbal communication condition.

Pair ID	Measurement Period (min:sec.msec)	Density (nods / min)	Mean Phase Difference (ms)	SD (ms)	Kurtosis
1	06:32.77	6.6	180	290	0.9
2	08:16.53	8.4	70	400	0.2
3	09:57.97	8.2	20	300	0.8
4	07:53.55	7.5	30	370	0.5
5	09:16.33	7.7	170	300	0.4
6	06:50.48	12.4	90	340	0.0
7	07:42.30	8.1	100	240	0.2
8	09:53.81	12.3	90	280	2.6
9	08:38.10	8.1	20	240	1.2
10	09:14.78	7.7	240	330	2.6
11	13:53.79	12.7	90	280	1.4
12	07:47.40	5.5	100	360	-0.2

Table 2. Result of the two-way verbal communication condition.

Pair ID	Measurement Period (min:sec.msec)	Density (nods / min)	Mean Phase Difference (ms)	SD (ms)	Kurtosis
1	08:07.40	9.9	50	300	1.0
2	08:09.21	12.9	-30	290	0.1
3	08:48.31	6.6	10	440	-0.7
4	09:05.49	5.3	60	420	-0.2
5	08:20.58	6.8	40	460	-0.2
6	08:52.16	12.1	-50	350	0.3
7	08:05.54	7.7	20	380	0.6
8	07:59.90	11.0	-20	250	1.5
9	08:55.64	8.0	0	360	0.3
10	09:49.78	9.5	-70	360	0.6
11	09:45.62	8.2	-60	320	0.6
12	08:38.31	5.6	10	460	-0.5
